# Prognostic Value of Local Treatment in Prostate Cancer Patients With Different Metastatic Sites: A Population Based Retrospective Study

**DOI:** 10.3389/fonc.2020.527952

**Published:** 2020-12-08

**Authors:** Shengming Jin, Jiaming Wei, Junjie Wang, Beihe Wang, Junlong Wu, Hualei Gan, Bo Dai, Xiaojian Qin, Guowen Lin, Yu Wei, Chen Yang, Yijun Shen, Yiping Zhu, Yao Zhu, Dingwei Ye

**Affiliations:** ^1^ Department of Urology, Fudan University Shanghai Cancer Center, Shanghai, China; ^2^ Department of Oncology, Shanghai Medical College, Fudan University, Shanghai, China; ^3^ Department of Pathology, Fudan University Shanghai Cancer Center, Shanghai, China; ^4^ Department of Urology, Huashan Hospital, Fudan University, Shanghai, China

**Keywords:** prostate cancer, distant metastasis, radical prostatectomy, brachytherapy, prognosis

## Abstract

**Purpose:**

Our study aims to examine the impact of definitive local therapy in prostate cancer patients with different metastatic sites.

**Methods:**

Totally, 5,849 patients diagnosed with metastatic prostate carcinoma from 2010 to 2014 were selected from Surveillance, Epidemiology, and End Results (SEER). Log-rank analyses, multivariable regression analysis, and Kaplan–Meier methods were used to assess prognostic impact of local treatment in patients with different metastatic sites. Survival curves and forest plots were also plotted to describe the prognostic value of definitive local therapy.

**Results:**

In our study, 159 patients received radical prostatectomy, and 62 received brachytherapy, while 5,628 did not receive local definitive local therapy. Survival analysis revealed that patients who received definitive local therapy had a better 5-year overall survival (OS) (P = 0.011) and cancer-specific survival (CSS) (P = 0.012). Multivariate regression analyses demonstrated that type of treatment was an independent prognostic indicator for OS (P = 0.011) and CSS (P = 0.012), along with age at diagnosis, chemotherapy, PSA level, and Gleason score. According to subgroup analysis, patients with bone metastasis or distant lymph node (LN) metastasis were significantly more likely to benefit from definitive local therapy. In addition, forest plots demonstrated that RP group had significant favorable OS and CSS in subgroups of younger age at diagnosis, T2–3 stage, N0–1 stage, Gleason score =7 or ≥8, bone metastasis, and distant LN metastasis.

**Conclusions:**

Our study suggested that local therapy improved survival in prostate cancer patients with bone or distant LN metastasis. Furthermore, patients who were at T2–3 stage or Gleason score ≥7 also significantly benefit from definitive local therapy.

## Introduction

Prostate carcinoma is one of the most common cancers in men (1). In 2016, it is estimated that more than three million men were currently prostate cancer patients in the United States, and many of them were diagnosed with metastasis (2). The prognosis of metastatic prostate cancer was comparatively poor, which caused significant mortality in prostate cancer patients (3).

Several studies have studied the distribution of metastatic sites in prostate cancer patients, and the most common ones were distant lymph nodes (LNs) and bones (4). Although many studies focused on the treatment of prostate cancer, the options for metastatic prostate cancer were still limited, and the standard therapy was androgen deprivation therapy combined with chemotherapy (3, 5). Shou et al. reported that patients with different metastatic sites seemed to have significant different prognosis in prostate cancer, which might have promising implications to make different clinical decisions to those patients (6).

Many studies reported that local therapy of the primary tumor, including radical prostatectomy (RP) or brachytherapy (BT) may improve survival outcomes of many metastatic cancers, such as bladder cancer (7, 8) and breast cancer (9–11). The role of definitive local therapy in the treatment of metastatic prostate cancer also received more and more attention. Some studies suggested that definitive local therapy could be a potential treatment option for metastatic prostate cancer (12–14). As for radiotherapy, the latest Systemic Therapy for Advanced or Metastatic Prostate cancer: Evaluation of Drug Efficacy (STAMPEDE) trial demonstrated an overall survival benefit (p = 0.007) for patients with a low metastatic burden. This was also suggested in the subgroup analysis of the HORRAD trial (15, 16). However, few studies have discussed the relationship between definitive local treatment and various metastatic sites of prostate cancer because of differences in treatment schemes and the small number of patients.

Comprehensive analysis on the prognostic impact of definitive local therapy for metastatic prostate cancer, especially for site-specific metastasis, is deficient. Therefore, the aim of our study was to examine the value of definitive local therapy in prostate cancer patients with different metastatic sites by population-based data from the SEER database. In addition, we also investigated the optimal candidates of definitive local therapy.

## Material and Methods

### Patients

Surveillance, Epidemiology, and End Results (SEER) database was used to identify patients’ information. The inclusion criteria for patients were as follows: 1) prostate cancer patients (the ICD-O-3 code for histology was 8,140) with distant metastasis at diagnosis (“M1” in the variable “Derived AJCC M”); 2) prostate cancer was only or first malignant tumor; 3) diagnosed from 2010 to 2014; and 4) patients with complete information on treatment (including surgery and radiotherapy), age at diagnosis and follow-up.

### Covariates and Survival Data

Baseline information included demographics factors (ethnicity, age at diagnosis, and marital status), disease status (Gleason score, PSA value, T stage, N stage, M stage, status of distant metastases: brain, lung, liver, bone, and distant lymph node) and therapy (radical prostatectomy, brachytherapy, and chemotherapy). In this study, RP represented the total dissection of the prostate. Incomplete local therapies like cryosurgery were not counted as RP. Factor for chemotherapy was simply categorized as patients who once did or did not receive chemotherapy once because of lack of detailed information in the SEER database.

In addition, survival information included overall survival (OS) and cancer-specific survival (CSS) in our study.

### Statistical Analysis

In this study, patients were classified into three subtypes according to their treatment history: not receiving radical prostatectomy or brachytherapy (NSR, N = 5628), receiving radical prostatectomy without brachytherapy (RP, N = 159), and receiving brachytherapy without radical prostatectomy (BT, N = 62). Clinicopathological variables of the three subgroups (NSR, RP, and BT) were collected for further analysis. Student’s t-test was used to compare continuous variable, and Pearson chi-square test or Fisher’s exact test was used to compare categorical variables. Log-rank test and Kaplan–Meier methods were used to perform survival analysis. Independent effect of clinical factors on OS and CSS was assessed by Cox’s multivariate analyses. Besides, forest plots and survival curves were also plotted.

All the statistical tests were conducted by SPSS (version 22.0, IBM. NY, USA). Kaplan–Meier survival curves were also plotted by SPSS. Venn diagram was drawn on the public website (http://bioinformatics.psb.ugent.be/webtools/Venn/). Besides, forest plots were depicted by R (version 3.5.1, Austria). All the statistical tests were double-sided, and P < 0.05 was considered statistically significant.

### Ethics Statement

Following the provisions of the Declaration of Helsinki (as revised in Fortaleza, Brazil, October 2013), this study was permitted by the Ethics Committee of Fudan University Shanghai Cancer Center. All the patients were from the public database. Therefore, informed consent was not needed.

## Results

### Patient Baseline Characteristics

**Figure 1:**
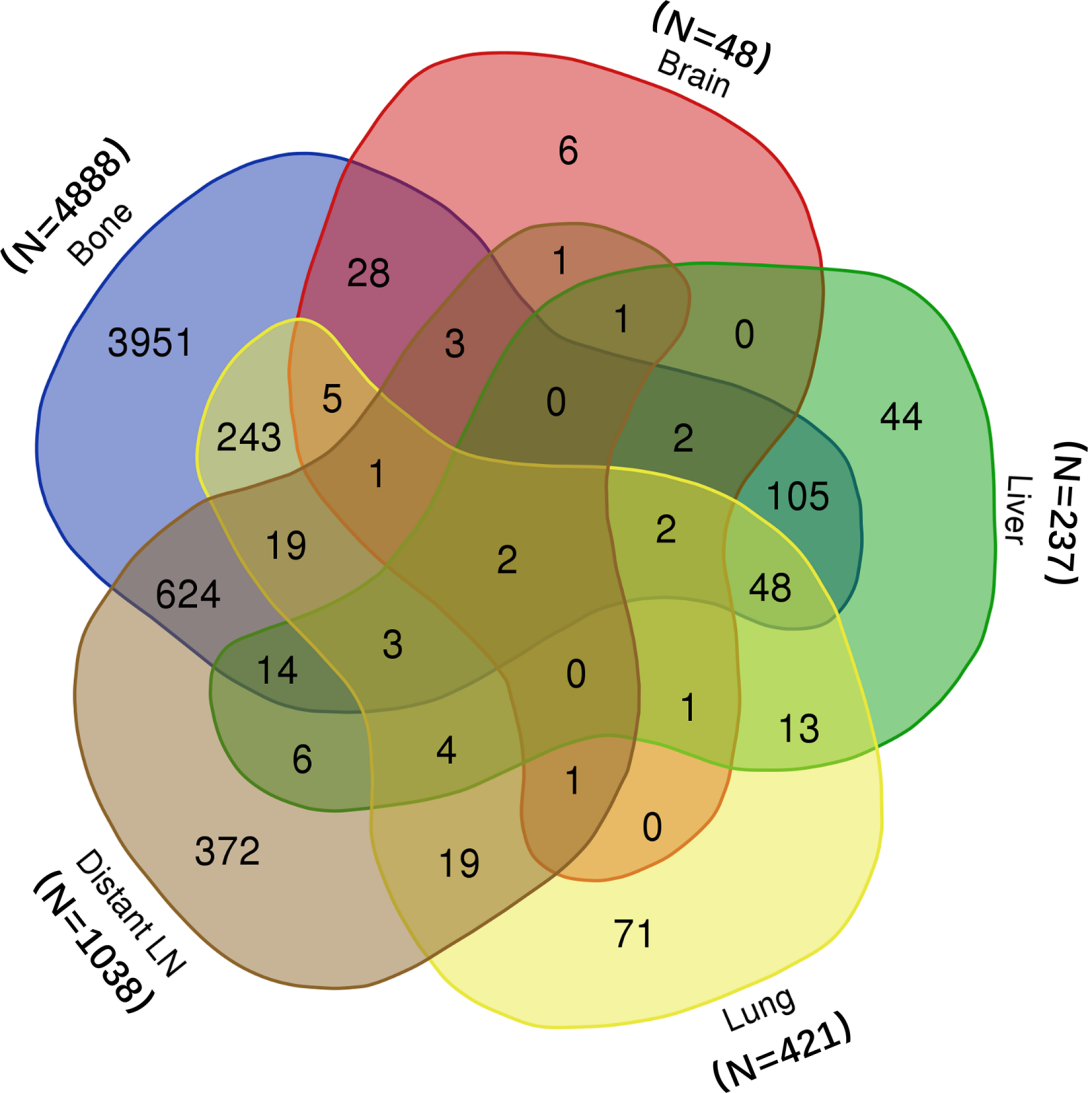
Venn diagram of distribution of metastatic sites.

In all, 5,849 patients from the SEER database were included in this study. Among them, 5,628 did not receive surgery or radiotherapy; 159 received radical prostatectomy, and 62 received brachytherapy. The metastatic information of all patients was shown in Venn plot (**Figure 1**). Most patients suffered from bone (n = 4,888) and distant LN (n = 1,038) metastasis. In addition, many patients had more than one organ metastasis.

Clinical information of these patients was demonstrated in **Table 1**. They were divided into NSR, RP, and BT cohorts. The median age at diagnosis was 70 for the NSR cohort (interquartile range: 62–78), 62 for the RP cohort (interquartile range: 57–67) and 65 for the BT cohort (interquartile range: 56–70). Patients belonging to the NSR cohort are more likely to be older at diagnosis, suffer from bone metastasis, and less likely to be married, compared to the other two groups. Also, there was a significant difference in T stage, M stage, PSA level, Gleason score among three groups. Otherwise, no significant difference in M stage, chemotherapy, brain, liver, lung, and distant LN metastasis was noted among the groups.

**Table 1:** Baseline characteristics of metastatic prostate cancer patients in the SEER database.

Characteristics	NSR (%, N = 5,628)	RP (%, N = 159)	P value^a^	BT (%, N = 62)	P value^b^	P value^c^
**Age at diagnosis**						
Mean(IQR)	70(62–78)	62(57–67)	<0.001	65(56–70)	<0.001	<0.001
<70	2,786(49.5)	137(86.2)	<0.001	44(71.0)	0.001	<0.001
≥70	2,842(50.5)	22(13.8)		18(29.0)		
**Marital status**			<0.001		0.09	<0.001
Married	3,030(53.8)	120(54.4)		42(67.7)		
Unmarried	2,132(37.9)	35(37.4)		16(25.8)		
Unknown	466(8.3)	4(8.1)		4(6.5)		
**Race**			0.203		0.683	0.401
White	4,114(73.1)	123(77.4)		43(69.4)		
Black	1,107(19.7)	22(13.8)		15(24.2)		
Other	341(6.1)	13(8.2)		4(6.5)		
Unknown	66(1.2)	1(0.6)		0(0)		
**AJCC T stage**			<0.001		0.002	<0.001
T1	1,469(26.1)	7(4.4)		30(48.4)		
T2	1,757(31.2)	51(32.1)		14(22.6)		
T3	616(10.9)	88(55.3)		3(4.8)		
T4	610(10.8)	9(5.7)		4(6.5)		
TX	1,176(20.9)	4 (2.5)		11(17.7)		
**AJCC N stage**			<0.001		0.02	<0.001
N0	3,035(53.9)	99 (62.3)		44(71.0)		
N1	1,452(25.8)	55 (34.6)		8(12.9)		
NX	1,141(20.3)	5 (3.1)		10(16.1)		
**AJCC M stage**			0.028		0.978	0.088
M1a	371 (6.6)	20 (12.6)		4(6.5)		
M1b	4,078 (72.5)	105 (66.0)		44(71.0)		
M1c	963 (17.1)	28 (17.6)		11(17.7)		
M1NOS	216 (3.8)	6 (3.8)		3(4.8)		
**Chemotherapy**			0.927		0.588	0.775
No evidence	5,284 (93.9)	149 (93.7)		60(96.8)		
Yes	344 (6.1)	10 (6.3)		2(3.2)		
**PSA, ng/ml**			<0.001		<0.001	<0.001
<10	429 (7.6)	86 (54.1)		21(33.9)		
10–19	451 (8.0)	30 (18.9)		7(11.3)		
20–29	348 (6.2)	12 (7.5)		4(6.5)		
≥30	3,813 (67.8)	20 (12.6)		24(38.7)		
Unknown	587 (10.4)	11 (6.9)		6(9.7)		
**Gleason Score**			<0.001		<0.001	<0.001
≤6	124 (2.2)	13 (8.2)		8(12.9)		
7	674 (12.0)	54 (34.0)		14(22.6)		
≥8	3,567 (63.4)	76 (47.8)		32(51.6)		
Unknown	1,263 (22.4)	16 (10.1)		8(12.9)		
**Bone metastases**			<0.001		0.037	<0.001
No evidence	740 (13.1)	45 (28.3)		14(22.6)		
Yes	4,888 (86.9)	114 (71.7)		48(77.4)		
**Brain metastases**			0.028		0.417	0.049
No evidence	5,580 (99.1)	155 (97.5)		61(98.4)		
Yes	48 (0.9)	4 (2.5)		1(1.6)		
**Liver metastases**			0.786		0.701	0.863
No evidence	5,391 (95.8)	153 (96.2)		60(96.8)		
Yes	237 (4.2)	6 (3.8)		2(3.2)		
**Lung metastases**			0.573		0.087	0.19
No evidence	5,207 (92.5)	149 (93.7)		61(98.4)		
Yes	421 (7.5)	10 (6.3)		1(1.6)		
**Distant LN metastases**			0.201		0.512	0.155
No evidence	4,590 (81.6)	136 (85.5)		53(85.5)		
Yes	1,038 (18.4)	23 (14.5)		9(14.5)		

a Comparing NSR cohort with RP cohort.

b Comparing NSR cohort with BT cohort.

c Comparing NSR cohort with the entire local therapy cohort.

### Multivariate Survival Analysis of all Patients

Cox multivariate analyses were conducted to evaluate clinical factors’ prognostic value for CSS and OS in the overall group (see **Table 2**). Comparing with the NSR group, patients who received RP or BT seemed to have better CSS (P = 0.012) and OS (P = 0.011). In addition, the results showed that age at diagnosis, marital status, race, T stage and M stage, chemotherapy, PSA level, and Gleason score were independent prognostic factors affecting OS and CSS. Nevertheless, no significant difference was seen among patients with different N stages. As for the metastatic sites, patients with bone and liver metastasis had significant worse prognosis, while there was no significant prognostic difference between patients with other metastasis (brain, lung and distant LN) or not (OS: P = 0.275; CSS: P = 0.112).

**Table 2:** Cox multivariate analyses of prognostic indicators for OS and CSS in the overall cohort.

Variables	Overall survival				Cancer-specific survival			
	HR	95%CI		P value	HR	95%CI		P value
**Age at diagnosis**	1.027	1.023	1.03	<0.001	1.022	1.018	1.026	<0.001
**Marital status**				<0.001				0.027
Married	Reference				Reference			
Unmarried	1.173	1.081	1.272	<0.001	1.111	1.014	1.217	0.024
Unknown	0.888	0.763	1.034	0.127	0.927	0.785	1.094	0.37
**Race**				<0.001				<0.001
White	Reference				Reference			
Black	1.081	0.98	1.192	0.119	1.087	0.975	1.212	0.131
Other	0.661	0.55	0.795	<0.001	0.66	0.537	0.811	<0.001
Unknown	0.31	0.161	0.597	<0.001	0.129	0.042	0.401	<0.001
**AJCC T stage**				<0.001				<0.001
T1	Reference				Reference			
T2	1.035	0.93	1.152	0.529	1.018	0.903	1.148	0.77
T3	0.953	0.822	1.105	0.526	0.986	0.837	1.161	0.863
T4	1.333	1.159	1.533	<0.001	1.369	1.174	1.597	<0.001
TX	1.177	1.028	1.348	0.018	1.154	0.992	1.343	0.064
**AJCC N stage**				0.275				0.112
N0	Reference				Reference			
N1	1.027	0.925	1.14	0.614	1.061	0.945	1.191	0.315
NX	1.088	0.982	1.206	0.108	1.128	1.006	1.266	0.04
**AJCC M stage**				0.004				0.007
M1a	Reference				Reference			
M1b	1.203	0.952	1.521	0.121	1.314	1.01	1.71	0.042
M1c	1.448	1.13	1.856	0.003	1.56	1.18	2.061	0.002
M1NOS	1.35	1.008	1.809	0.044	1.437	1.034	1.999	0.031
**Chemo therapy**				0.037				0.003
No evidence	Reference				Reference			
Yes	1.178	1.01	1.374	0.037	1.283	1.089	1.51	0.003
**Type of treatment**				0.011				0.012
NSR	Reference				Reference			
RP	0.604	0.418	0.872	0.007	0.564	0.372	0.856	0.007
BT	0.722	0.458	1.139	0.161	0.709	0.425	1.183	0.188
**PSA, ng/ml**				<0.001				<0.001
<10	Reference				Reference			
10–19	0.98	0.795	1.207	0.846	0.955	0.757	1.205	0.696
20–29	1.005	0.802	1.259	0.964	0.925	0.716	1.195	0.551
≥30	1.321	1.127	1.548	0.001	1.289	1.082	1.536	0.005
Unknown	1.351	1.115	1.638	0.002	1.276	1.029	1.582	0.026
**Gleason Score**				<0.001				<0.001
≤6	Reference				Reference			
7	0.981	0.715	1.345	0.903	1.124	0.765	1.652	0.55
≥8	1.475	1.097	1.983	0.01	1.799	1.253	2.584	0.001
Unknown	1.867	1.373	2.538	<0.001	2.319	1.596	3.369	<0.001
**Bone metastases**				0.004				0.004
No evidence	Reference				Reference			
Yes	1.248	1.074	1.449	0.004	1.278	1.081	1.51	0.004
**Brain metastases**				0.207				0.365
No evidence	Reference				Reference			
Yes	1.251	0.883	1.773	0.207	1.199	0.81	1.776	0.365
**Liver metastases**				<0.001				<0.001
No evidence	Reference				Reference			
Yes	2.012	1.698	2.383	<0.001	2.238	1.865	2.685	<0.001
**Lung metastases**				0.575				0.846
No evidence	Reference				Reference			
Yes	0.956	0.817	1.119	0.575	0.983	0.827	1.169	0.846
**Distant LN metastases**				0.231				0.137
No evidence	Reference				Reference			
Yes	1.082	0.951	1.23	0.231	1.113	0.967	1.28	0.137

### Prognostic Significance of Definitive Local Therapy in Patients With Metastatic Prostate Cancer

**Figure 2:**
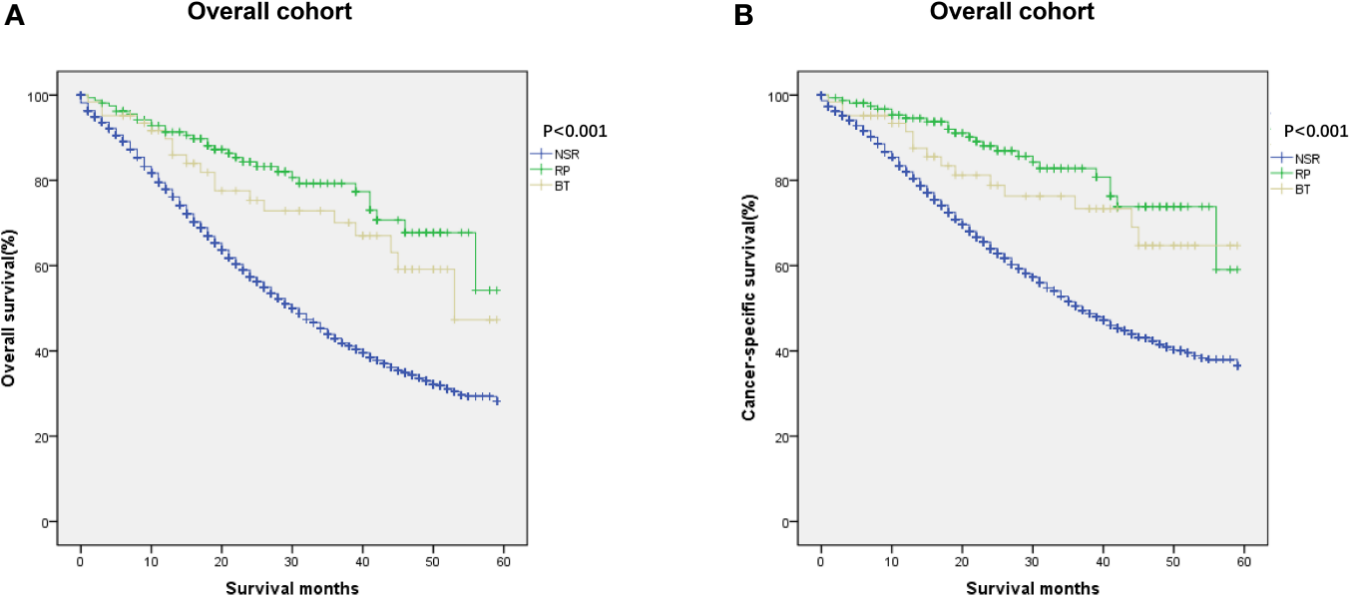
Kaplan–Meier curves of **(A)** overall and **(B)** cancer-specific survival in NSR, RP, and BT groups were performed in the overall cohort (n = 455).

We then analyzed whether local therapy could improve outcomes for metastatic prostate cancer patients. Multivariate analyses revealed that type of treatment was a prognostic factor affecting OS and CSS among all patients (**Table 2**). Therefore, we investigated the prognostic significance of definitive local therapy in overall cohort using Kaplan–Meier curves and log-rank test. The results were presented in **Figure 2**, showing significant better survival in OS and CSS for the RP and BT groups (OS: P < 0.001; CSS: P < 0.001).

**Figure 3:**
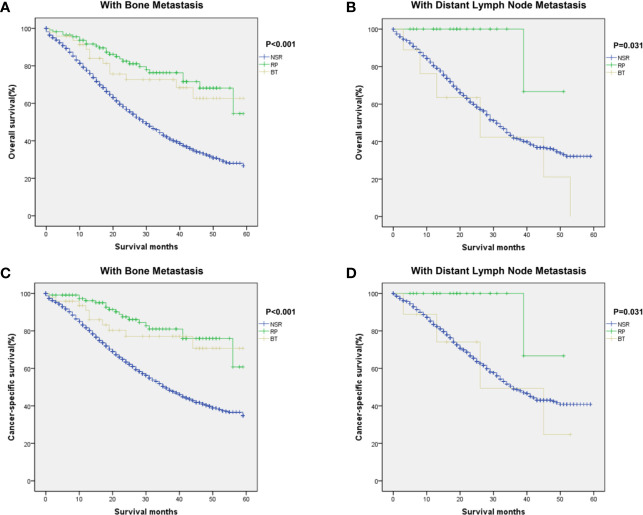
Kaplan–Meier curves of overall and cancer-specific survival in the NSR, RP, and BT groups were performed in patients with bone metastases **(A, C)** and distant lymph nodes metastases **(B, D)**.

In this study, we further analyzed if metastatic sites of prostate cancer affect prognostic outcomes among the three groups. For patients with bone metastasis, Kaplan–Meier curves showed significant difference in OS and CSS between patients in different treatment groups (P < 0.001 for OS, P < 0.001 for CSS, **Figures 3A, C**). RP and BT groups also conferred a significant survival advantage in patients with distant metastatic LN (OS: P = 0.031; CSS: P = 0.031, **Figures 3B, D**).

We then performed subgroup analyses to compare survival differences in different Gleason scores groups and T stage groups. For patients with Gleason score =7 or Gleason score ≥8, patients who received RP or BT showed better survival outcome than patients who did not received local therapy (**Supplementary Figures 1B, C, E, F**). However, no significant difference of OS and CSS was found in subgroup Gleason score ≤6 (**Supplementary Figures 1A, D**), indicating that definitive local therapy may not be recommended for patients with low Gleason scores. For patients with different T stages, T2 and T3 patients had higher OS and CSS in the RP or BT cohort (**Supplementary Figures 2B, C, F, G**). Patients in T1 stage only had a significant difference in OS (**Supplementary Figures 2A, E**). Patients in T4 stage did not have a significant difference in OS or CSS (**Supplementary Figures 2D, G**).

**Figure 4:**
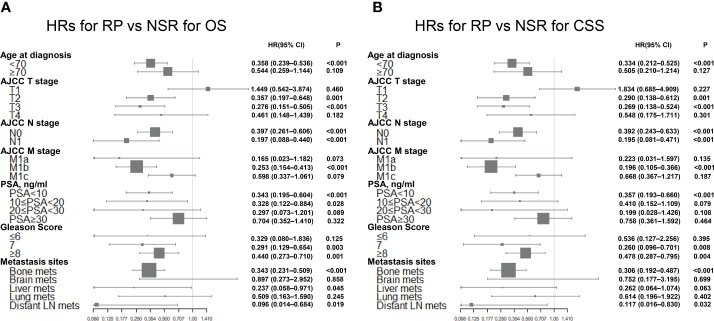
Forest plots summarizing the HRs and 95% CIs of **(A)** overall and **(B)** cancer-specific survival for RP *versus* NSR in subgroup analyses.

Moreover, Cox’s multivariate analyses were carried out, and a forest plot was made (**Figure 4**) to better compare the prognostic impact of definitive local therapy. No significant difference in OS and CSS was found between the NSR and RP cohorts for most of the subgroups. However, the RP group had significant favorable OS and CSS in the subgroups of younger age at diagnosis, T2–3 stage, N0–1 stage, M1b stage, Gleason score =7 or ≥8, bone metastasis, and distant LN metastasis. It is worth noting that no significant difference exists in the OS and CSS for subgroups of T1 stage between RP and NSR cohorts. Subgroup analysis for BT *versus* NSR was not presented due to lack of eligible patients in the BT group.

## Discussion

Metastasis is common in prostate cancer patients and is often correlated with poor outcome. Management strategy of metastatic prostate cancer is developing over the last few decades. Newly diagnosed metastatic patients normally have a median survival of more than 40 months (5). Some studies have suggested that distant metastasis pattern has independent prognostic value. For example, Shou and his colleague revealed that patients with liver metastasis have worse survival outcome than bone, brain, and lung metastases (6). In our study, using the metastatic prostate cancer cohort from the SEER database, we retrospectively studied the effect of local treatment in prostate cancer patients with different sites of metastasis and proved that definitive local therapy improved survival in patients with bone or LN metastasis. Moreover, we for the first time pointed out that Gleason score and T stage were both independent predictors for the prognosis of metastatic prostate cancer patients receiving definitive local therapy.

The therapeutic value of local therapy on patients’ prognosis has been discussed in many metastatic cancers (7–11, 17, 18). Recently, local therapy was considered a standard therapy for metastatic breast cancer patients (19). Adoption of local therapy in other metastatic cancers has brought us ideas to explore its value in metastatic prostate cancer. However, some studies previously claimed that if the tumor had spread across the capsule of the prostate, definitive local therapy to the tumor could not control the development of tumor or improve prognosis, but only alleviate local symptoms (20, 21). Therefore, definitive local therapy is rarely considered as a conventional treatment method in prostate cancer management (3, 5). Recently, with the application of definitive local therapy in other metastatic cancers, some researches aimed at its therapeutic value in metastatic prostate cancer. Michael et al. suggested that cytoreductive prostatectomy, when combined with multimodal management, should be considered in metastatic prostate cancer patients, which may improve the prognosis (22). Stephen et al. found a survival benefit for local treatment in metastatic prostate cancer patients in a retrospective study (14). Based on a large patient cohort from the SEER database, our study investigated which types of metastatic prostate patients could benefit from definitive local therapy based on the large cohort of metastatic prostate patients from the SEER database. It might provide new insight into clinical management of metastatic prostate cancer patients and help classify patients with various prognoses into different groups.

In this study, we divided patients into different subgroups according to patients’ clinical features and discussed which subgroup of patients was more suitable for definitive local therapy. In the overall cohort, patients who received RP or BT had significant 5-year OS and CSS. This result was in line with many previous researches on other types of cancer. Furthermore, in this study, we originally demonstrated that prostate cancer patients with bone or distant lymph node metastasis were associated with significant better prognosis after definitive local therapy, while the same results did not occur in liver, brain or lung metastasis. This situation might be due to the factor that the brain, liver, and lung are important organs and metastasis could more easily induce organ failures in these sites, which cannot be controlled by definitive local therapy. Additionally, definitive local therapy turned out to be an independent prognostic factor in subgroups of T2, T3 stage, Gleason score =7 or Gleason score ≥8. These results indicated that metastatic prostate cancer patients with clinical features above may benefit from definitive local therapy.

Recently, the prognostic value of definitive local therapy has been discussed in metastatic prostate cancer and many other cancers. However, in the 2019 EAU guideline on prostate cancer, definitive local therapy is still not recommended as routine treatment for all metastatic prostate cancer patients because of the lack of evidence from large ongoing trials (5). Our study pointed out that metastatic prostate cancer patients with distant lymph node or bone metastasis would have better outcome after definitive local therapy. Moreover, patients who were at T2–3 stage and Gleason score =7 or ≥8 also significantly benefited from definitive local therapy. Such results indicated that definitive local therapy could possibly apply to highly selected groups of metastatic prostate cancer patients. Furthermore, by reducing the general tumor burden, local treatment could be used as a supplement for a comprehensive treatment scheme to improve tumor control (12). To verify the therapeutic value of definitive local therapy, more high-quality randomized controlled trials are in need.

This study still has several limitations. Firstly, selection biases would inevitably exist due to missing detailed information in the SEER database, like performance status and comorbidities. It’s reasonable that patients who have better physical condition will have a higher chance to receive RP or BT. However, some other studies based on the SEER database have also reported this disadvantage. They confirmed that it was impossible that this unadjusted confounding factor alone contributed to the highly significant prognostic value of RP or BT in highly selected groups (23–27). Secondly, the SEER database only had information of five specific organs of metastases, which could not include all types of metastasis developed in prostate cancer patients. Thirdly, there was a lack of detailed information about chemotherapy and androgen deprivation therapy in the SEER database, which could influence patients’ prognosis. Lastly, due to the relatively small number of patients receiving BT, we could not perform subgroup analysis for BT *versus* NSR.

## Conclusion

This study suggested that local therapy improved survival in prostate cancer patients with bone or distant LN metastasis. Moreover, patients who were at T2–3 stage and Gleason score ≥7 also significantly benefit from definitive local therapy. More high-quality randomized controlled trials are still required to verify the therapeutic value of definitive local therapy.

## Data Availability Statement

All datasets generated for this study are included in the article/**Supplementary Material**.

## Ethics Statement

This study was approved by the Research Ethics Committee of Shanghai Cancer Center, Fudan University, China, according to the provisions of the Declaration of Helsinki

## Author Contributions

SJ, JMW, and JJW analyzed the data and drafted the manuscript. BW, HG, and JLW helped interpreted the data. BD, XQ, and GL prepared all figures. YW, CY, and YS edited all tables. YPZ, YZ, and DY designed the study. All authors contributed to the article and approved the submitted version.

## Funding

This work was funded by the National Natural Science Foundation of China (No. 81772706 & 81972375). The funding source provided financial support for the study and did not have any other involvement in this study.

## Conflict of Interest

The authors declare that the research was conducted in the absence of any commercial or financial relationships that could be construed as a potential conflict of interest.

## Supplementary Material

Supplementary Figure 1 Kaplan–Meier curves of overall and cancer-specific survival in NSR, RP and BT groups were performed in patients with Gleason Score ≤6 **(A, D)**, Gleason Score =7 **(B, E)** and Gleason Score ≥8 **(C**, **F)**.

Supplementary Figure 2 Kaplan–Meier curves of overall and cancer-specific survival in the NSR, RP, and BT groups were performed in patients with T1 **(A, E)**, T2 **(B, F)**, T3 **(C, G)** and T4 **(D, H)** stage.
