# Supplementary figures and images for: Prognostic Value of Local Treatment in Prostate Cancer Patients With Different Metastatic Sites: A Population Based Retrospective Study
Source: Front Oncol. 2020 Dec 8;10:527952. doi: 10.3389/fonc.2020.527952 (PMC7753115; doi:10.3389/fonc.2020.527952)

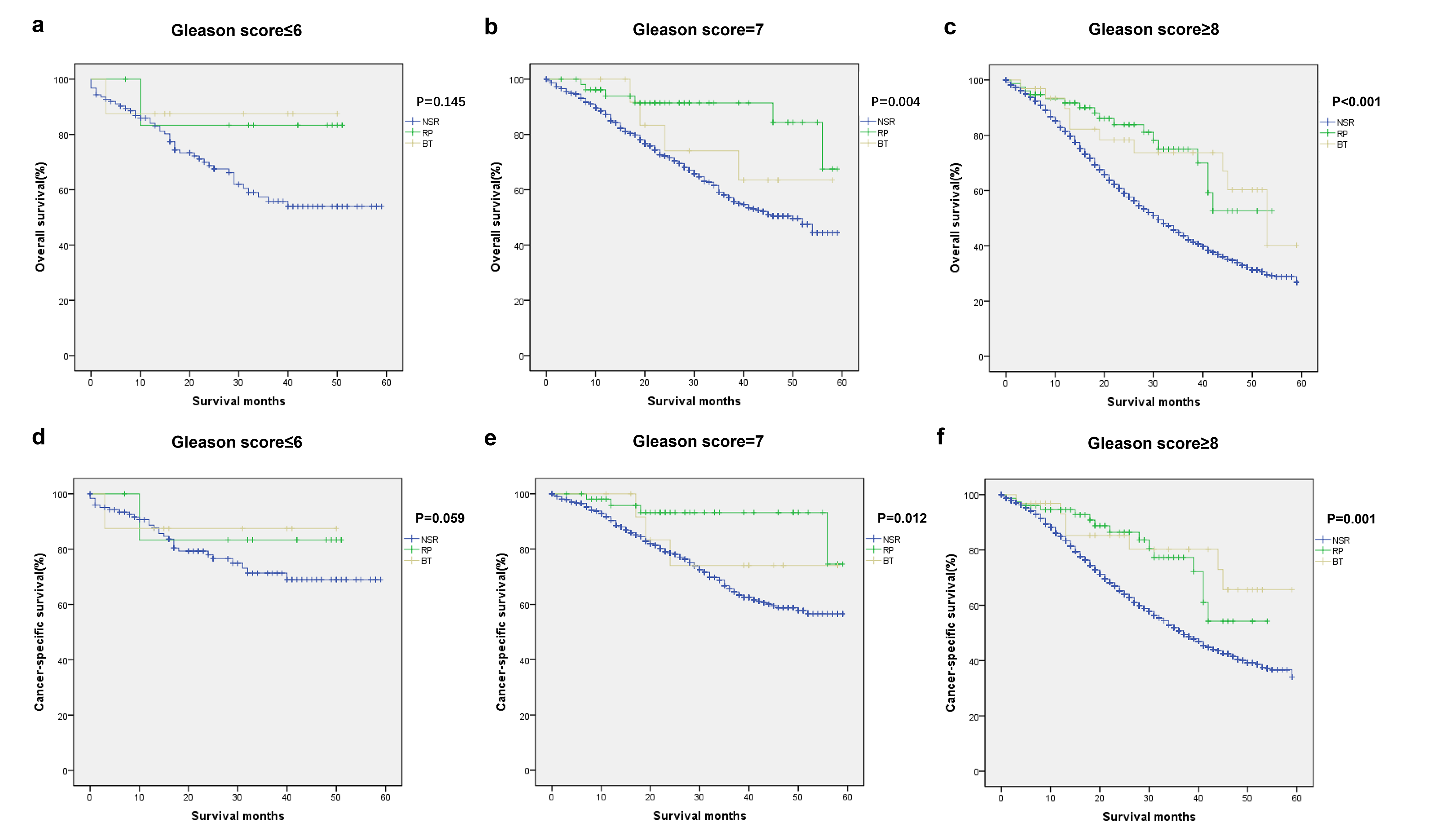

Supplement: Supplementary Figure 1 — Kaplan–Meier curves of overall and cancer-specific survival in NSR, RP and BT groups were performed in patients with Gleason Score ≤6 (A, D), Gleason Score =7 (B, E) and Gleason Score ≥8 (C, F). [file Image_1.tif]

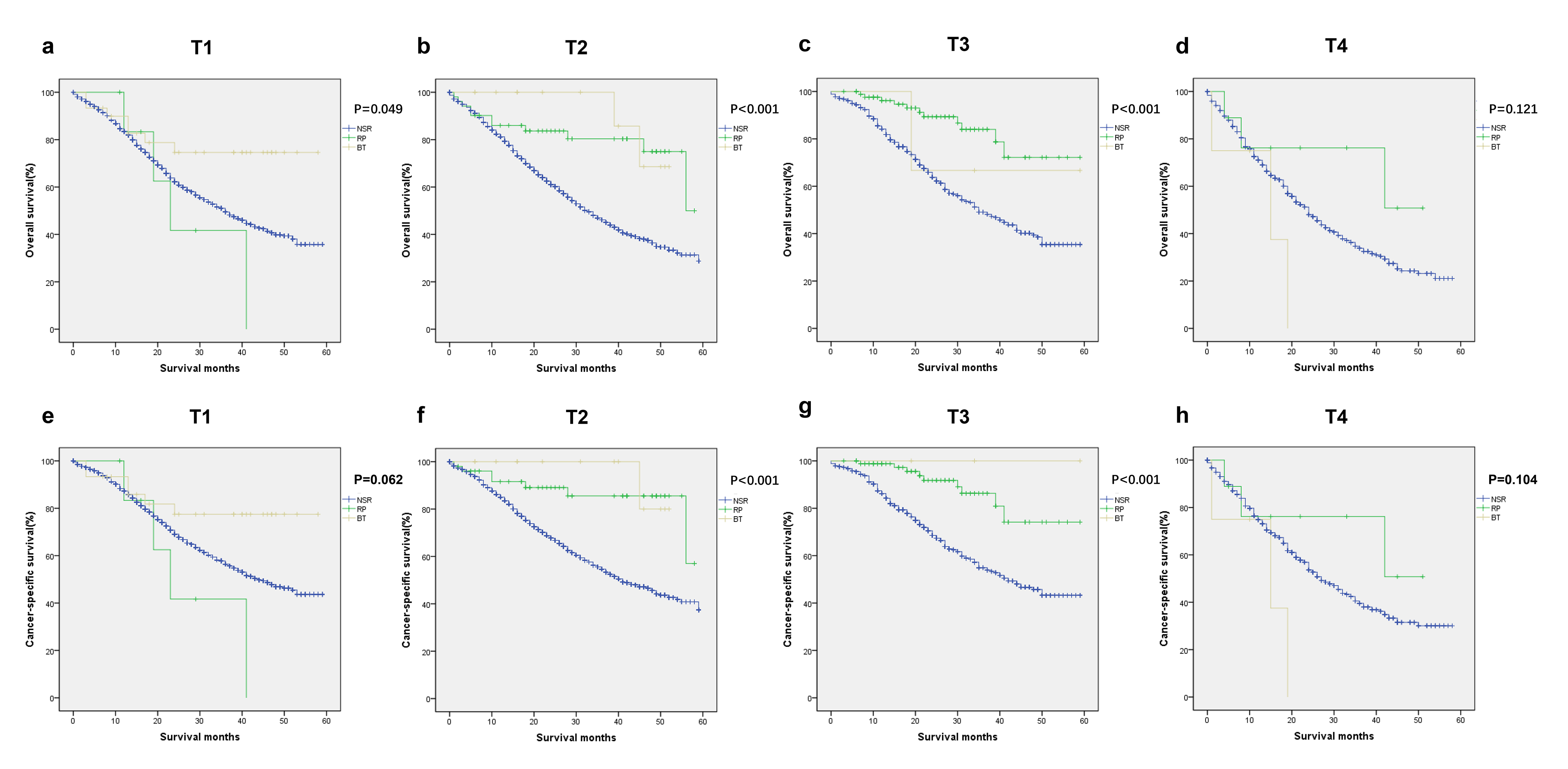

Supplement: Supplementary Figure 2 — Kaplan–Meier curves of overall and cancer-specific survival in the NSR, RP, and BT groups were performed in patients with T1 (A, E), T2 (B, F), T3 (C, G) and T4 (D, H) stage. [file Image_2.tif]
